# Gene therapy in the putamen for curing AADC deficiency and Parkinson's disease

**DOI:** 10.15252/emmm.202114712

**Published:** 2021-08-23

**Authors:** Paul Wuh‐Liang Hwu, Karl Kiening, Irina Anselm, David R Compton, Takeshi Nakajima, Thomas Opladen, Phillip L Pearl, Agathe Roubertie, Thomas Roujeau, Shin‐ichi Muramatsu

**Affiliations:** ^1^ Department of Medical Genetics and Pediatrics National Taiwan University Hospital Taipei Taiwan; ^2^ Department of Pediatrics National Taiwan University College of Medicine Taipei Taiwan; ^3^ Division of Stereotactic Neurosurgery Department of Neurosurgery University of Heidelberg Medical Center Heidelberg Germany; ^4^ Department of Neurology Boston Children’s Hospital Harvard Medical School Boston MA USA; ^5^ Preclinical Development (Gene Therapy) PTC Therapeutics South Plainfield NJ USA; ^6^ Department of Neurosurgery Jichi Medical University Tochigi Japan; ^7^ Jichi Medical University Hospital Rehabilitation Center Tochigi Japan; ^8^ Division of Child Neurology and Metabolic Disorders University Children’s Hospital Heidelberg Germany; ^9^ Epilepsy and Clinical Neurophysiology William G. Lennox Chair and Professor of Neurology Harvard Medical School Boston Children’s Hospital Boston MA USA; ^10^ Pediatric Neurology Department INM, INSERM, CHU Montpellier University of Montpellier Montpellier France; ^11^ Department of Neurosurgery Gui‐de‐Chauliac Hospital Montpellier University Hospital Montpellier France; ^12^ Institute of Neurosciences University Hospital of Montpellier Montpellier France; ^13^ Division of Neurological Gene Therapy Jichi Medical University, Shimotsuke Tochigi Japan; ^14^ Center for Gene & Cell Therapy The Institute of Medical Science The University of Tokyo Tokyo Japan

**Keywords:** AADC deficiency, aromatic l‐amino acid decarboxylase, dopamine, gene therapy, putamen, Genetics, Gene Therapy & Genetic Disease, Neuroscience

## Abstract

This commentary provides an overview of the putamen as an established target site for gene therapy in treating aromatic l‐amino acid decarboxylase (AADC) deficiency and Parkinson’s disease, two debilitating neurological disorders that involve motor dysfunction caused by dopamine deficiencies. The neuroanatomy and the function of the putamen in motor control provide good rationales for targeting this brain structure. Additionally, the efficacy and safety of intraputaminal gene therapy demonstrate that restoration of dopamine synthesis in the putamen by using low doses of adeno‐associated viral vector serotype 2 to deliver the hAADC gene is well tolerated. This restoration leads to sustained improvements in motor and nonmotor symptoms of AADC deficiency and improved uptake and conversion of exogenous l‐DOPA into dopamine in Parkinson’s patients.

## Graphical abstract

This Commentary provides an overview of putamen‐targeted gene therapies for treating aromatic l‐amino acid decarboxylase (AADC) deficiency and Parkinson’s disease.

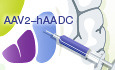

## Gene therapy for treating neurological disorders

Neurological disorders are considered some of the most difficult disorders to treat owing to the complexity of the central nervous system (CNS) and the blood–brain barrier (BBB) which limits the uptake of drugs or other therapeutic agents into the CNS. Innovative new therapies, such as gene therapy, antisense oligonucleotides, small molecules, and other advanced therapeutical medicinal products, are increasingly being developed and approved to treat a variety of neurological disorders. Among these, gene therapy is particularly promising as it targets the underlying causes of disease and can therefore provide a real cure instead of life‐long treatment (Piguet *et al*, 2017). Nonetheless, gene therapy using viral vectors faces the same challenge posed by the BBB. Direct delivery of vector to the appropriate brain area by stereotactic surgery circumvents this barrier.

For diseases that affect broad areas of the brain or the spinal cord, such as spinal muscular atrophy, therapeutic genes can be administered systemically either via intravenous delivery or intracerebroventricular or intrathecal injection into the cerebrospinal fluid (CSF). Although some viral vectors for gene therapy can cross the BBB, they still must be administered in large doses to successfully transfect cells in the CNS, which can cause acute liver failure and dorsal root ganglia toxicity. Similarly, CSF‐based delivery is associated with vector leakage into the bloodstream and subsequent off‐target tissue transduction, particularly in the liver. Systemically administered gene therapies are also susceptible to neutralizing antibodies against wild‐type adeno‐associated virus (AAV), which can limit transfection and elicit delayed immune responses against transfected liver cells that often require corticosteroid treatment either prophylactically or after treatment (Piguet *et al*, 2017).

Intraparenchymal delivery directly to target regions in the brain through stereotactic injections therefore appears to be the most appropriate method for treating various diseases, including Parkinson’s disease (PD) and aromatic l‐amino acid decarboxylase (AADC) deficiency, in which the main dysfunction is relatively confined to particular brain areas (Piguet *et al*, 2017). Intraputaminal dosing in non‐human primates showed distribution to the target with limited leakage of the vector to other brain regions. Overall, intraparenchymal administration is associated with favorable safety/tolerability profiles as it does not require large doses, it limits the risk of potential dorsal root ganglion nerve cell toxicity and liver‐related immunogenicity, thereby reducing or eliminating the need for corticosteroids, and it directly targets the brain areas of interest (Piguet *et al*, 2017).

## The putamen as a target site for gene therapy

**Figure 1:**
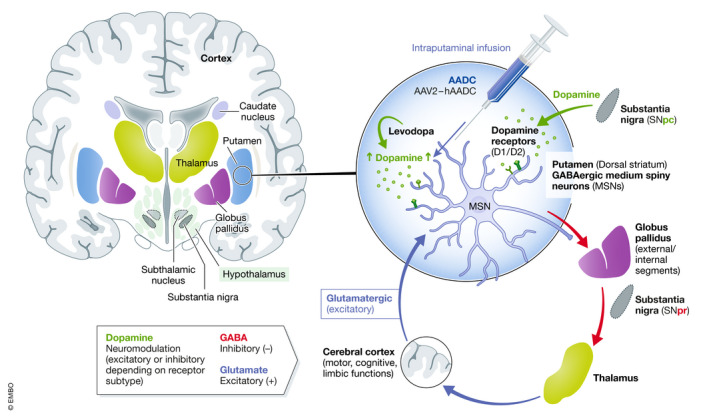
Simplified circuit diagram illustrating the key afferent and efferent connections of the dorsal striatum (putamen) and their role in control of motor, cognitive, and limbic functions Dopaminergic inputs to the putamen originate from the SNpc. The dopaminergic terminals release dopamine, which modulates the output of the postsynaptic MSNs in the putamen via D1 or D2 receptor activation. MSNs connect with different parts of the cerebral cortex indirectly via their connections with other basal ganglia nuclei (globus pallidus and SNpr) and thalamus. By exerting their inhibitory effects via these indirect connections (cortical and subcortical loops), the MSNs of the putamen control various functions (motor, cognitive, and limbic). Hence, dopamine, by modulating MSN function, exerts an important neuromodulatory effect on motor, cognitive, and limbic functions. The seat of this neuromodulation is in the striatum (caudate nucleus and putamen). MSN, medium spiny neuron; SNpc, substantia nigra pars compacta; SNpr, substantia nigra pars reticulata.

PD and AADC deficiency are two debilitating neurological disorders that involve motor dysfunction caused by dopamine (DA) deficiencies. DA is a key monoamine neurotransmitter that acts within the striatum to modulate the output of neurons in regions of the brain that are involved in voluntary motor movements, learning and memory, cognition, and emotion. The putamen is integral for planning and executing normal motor functions. Here, we highlight critical components of the striatal connections for motor control to support our rationale for choosing the putamen as a target site for gene therapy against neurological disorders that involve dopaminergic system dysfunction (Fig 1) (Haber, 2016).

The basal ganglia consist of the striatum (dorsal striatum [caudate nucleus and putamen] and ventral striatum [nucleus accumbens and olfactory tubercle]), globus pallidus, ventral pallidum, substantia nigra (SN), and the subthalamic nucleus. Each component has a complex anatomical and neurochemical organization, and they are highly interconnected to cooperatively modulate motor movements (Haber, 2016). The putamen is an important hub for the cortico‐basal ganglia network (Onuki *et al*, 2021): It receives substantial input from the primary and secondary somatic sensory cortices in the parietal lobe, the secondary visual cortices in the occipital and temporal lobes, the premotor and motor cortices in the frontal lobe, and the auditory association areas in the temporal lobe (Haber, 2016; Onuki *et al*, 2021).

The striatum is predominantly composed of γ‐aminobutyric acid (GABA)‐ergic medium spiny neurons (MSNs), which are the largest basal ganglia output sources that project primarily to the globus pallidus and the substantia nigra pars reticulata (SNpr) (Haber, 2016; Onuki *et al*, 2021). These pathways connect the putamen to the upper motor neurons in the cortex and midbrain (Haber, 2016). The globus pallidus innervates the thalamus, which sends projections directly to upper motor neurons of the cortex, completing a loop that originates in multiple cortical areas and terminates in frontal lobe motor areas (Haber, 2016; Onuki *et al*, 2021).

## AADC gene therapy to the putamen

In neurological disorders involving dopaminergic system dysfunction, such as PD and AADC deficiency, the putamen is directly impacted by the loss of DA synthesis in the striatum (Muramatsu *et al*, 2010; Onuki *et al*, 2021) and therefore a prime target for gene therapy. Furthermore, being a large structure in the forebrain, the putamen is more easily accessible via surgery than other potential sites, such as the SN. Here, we focus on PD and AADC deficiency, as these share aspects of their pathophysiologies; indeed, the studies on intraputaminal infusion of gene therapy against PD served as a basis for developing the therapy against AADC deficiency. The therapeutic gene in both cases is the *dopa decarboxylase* (*DDC*) gene which encodes AADC (EC 4.1.1.28). This enzyme catalyzes the biosynthesis of essential monoamine neurotransmitters by decarboxylating 5‐hydroxytryptophan to serotonin and l‐3,4‐dihydroxyphenylalanine (l‐DOPA) to DA (Hwu *et al*, 2012).

PD is characterized by gradual loss of dopaminergic neurons in the SNpc, which leads to DA depletion in motor portions of the putamen. Exogenous l‐DOPA, levodopa, is currently the most effective medication. However, as PD progresses, AADC enzyme levels decline because of continual degradation of the dopaminergic SNpc striatal nerve terminals; thus, increased levodopa dosing is required to maintain the desired clinical response. AADC deficiency is caused by autosomal recessive inherited pathogenic mutants of the *DDC* gene (7p12.2‐p12.1, chr7[hg38]: 50,458,436–50,565,460), which prevents the conversion of l‐DOPA into DA that leads to decreased availability of serotonin and dopamine in the presynaptic and synaptic cleft, as well as a deficiency of catecholamines (Hwu *et al*, 2012). The rationale for intraputaminal AADC gene therapy is to deliver a functional copy of the human *DDC* gene (hAADC) directly to striatal regions impacted by the disease, which subsequently leads to increased conversion of levodopa to DA in targeted striatal neuronal cell bodies, which do not degenerate (Muramatsu *et al*, 2010).

A 2002 study by Muramatsu and colleagues in a non‐human primate model of advanced PD showed that intraputaminal gene delivery of dopamine‐synthesizing enzymes, including AADC, resulted in robust transgene expression and restoration of putaminal DA levels (Muramatsu *et al*, 2002). The treatment sufficiently restored motor functions, with remarkable improvement in manual dexterity and amelioration of tremor, bradykinesia, and muscle rigidity. Long‐term recovery and persistent transgene expression were documented up to 15 years after therapy (Sehara *et al*, 2017). Transduced neurons were broadly distributed across the putamen with no signs of cytotoxicity or Lewy body pathology (Sehara *et al*, 2017). Microdialysis showed increased extracellular DA in the transduced putamen during concomitant peripheral levodopa administration (Muramatsu *et al*, 2002). These findings demonstrate the long‐term safety and efficacy of intraputaminal delivery of AADC gene therapy in PD (Sehara *et al*, 2017).

The safety, pharmacodynamics, and preliminary efficacy of adeno‐associated viral vector serotype 2 (AAV2) mediated AADC gene therapy to the putamen were further evaluated in 3 phase 1 studies in 31 patients with PD: two in the USA (Christine *et al*, 2009; Mittermeyer *et al*, 2011; Christine *et al*, 2019) and one in Japan (Muramatsu *et al*, 2010). The procedure and treatment were generally well tolerated. Intracranial hemorrhages were reported in four patients (Muramatsu *et al*, 2010; Mittermeyer *et al*, 2011), and one patient developed deep vein thrombosis and pulmonary embolism, followed by atrial fibrillation (Christine *et al*, 2019). These adverse events were considered related to the surgical procedure rather than gene transduction (Muramatsu *et al*, 2010; Mittermeyer *et al*, 2011; Christine *et al*, 2019).

Given the demonstrated efficacy and safety of intraputaminal delivery of AADC gene therapy in patients with PD, the next step was to apply this approach to treat AADC deficiency (Hwu *et al*, 2012). The disorder is characterized by global developmental delay and severe extrapyramidal movement disorder with hypo‐ or akinesia, dystonia, and trunk‐emphasized muscular hypotonia. Oculogyric crises (OGCs)—involuntary eye (upward) movements—and further involuntary movements of other body parts (face, neck, trunk, or extremities) are also typical symptoms (Hwu *et al*, 2012; Onuki *et al*, 2021). No causal therapy has been approved for treating AADC deficiency, and the success of conventional drug treatment using combinations of vitamin B_6_, dopamine agonists, and monoamine oxidase inhibitors is very limited, especially in severe cases. Although other surgical strategies, such as deep brain stimulation, have shown efficacy in alleviating clinical symptoms in PD, these treatment options have not been evaluated in AADC deficiency. Thus, therapies that provide lasting and clinically meaningful improvements in motor development and cognitive function are urgently needed.

Although the pathophysiologies of PD and AADC deficiency differ—the former is a neurodegenerative disease, while the latter is an enzyme deficiency—lessons from intraputaminal gene therapy in PD were crucial in developing its applications in AADC deficiency (Table 1). Patients with PD receive exogenous l‐DOPA, which is converted to DA. In contrast, most patients with AADC deficiency lack the AADC enzyme from birth, which leads to excessive endogenous l‐DOPA levels. AADC gene therapy aims to convert endogenous l‐DOPA into DA (Muramatsu *et al*, 2002; Muramatsu *et al*, 2010).

**Table 1:** Brief overview of PD and AADC deficiency disease characteristics and gene therapy.

	PD	AADC deficiency
Disease characteristics		
Age at disease onset, mean	60 years	2.7 months
Pathophysiology	Striatal DA deficiency Due to degeneration of dopaminergic neurons in SNpc	Striatal DA deficiency Due to inborn AADC deficiency of dopaminergic neurons in the SNpc
Dopaminergic neurons	Degenerate early in disease	Remain intact
Gene therapy		
Levodopa	Levodopa treatment	No levodopa treatment
Dosing	3.0 × 10^11^–5.4 × 10^11^ vg	1.8 × 10^11^–2.4 × 10^11^ vg
Mechanism of action	Transduction of MSNs	Transduction of MSNs and/or monoenzymatic/dienzymatic neurons

AADC, aromatic l‐amino acid decarboxylase; DA, dopamine; MSN, medium spiny neuron; PD, Parkinson's disease; SNpc, substantia nigra pars compacta.

The efficacy and safety of AAV2 gene therapy via intraputaminal bilateral infusions in children with AADC deficiency (*n* = 20) have been investigated in 3 clinical trials conducted by different investigators (Hwu *et al*, 2012; Chien *et al*, 2017; Kojima *et al*, 2019). The effective doses for gene therapies used in studies of adults with PD (3 × 10^11^ and 5.4 × 10^11^ vg) were the basis for calculating the dose range used in subsequent studies of AADC gene therapy in children (Christine *et al*, 2009; Muramatsu *et al*, 2010; Mittermeyer *et al*, 2011; Hwu *et al*, 2012).

Prior to gene therapy, nearly all patients in these studies had characteristic manifestations of severe AADC deficiency, including hypokinesia, dystonia, oculogyric crisis, emotional instability, and increased sputum; were bed ridden; and had not achieved full head control or more advanced motor functions (Hwu *et al*, 2012; Chien *et al*, 2017; Kojima *et al*, 2019). The only exception was the Kojima *et al* study, in which one patient with moderate severity could walk albeit with support (Chien *et al*, 2017; Kojima *et al*, 2019).

Patients received 1.8 × 10^11^ vg or 2 × 10^11^ vg AAV2‐hAADC infused bilaterally into the putamen. The procedure and treatment were well tolerated in all cases. After gene transfer, all patients showed improvements in OGC symptoms, emotional stability, and motor function including good or partial head control. Some patients were able to sit and stand with support and began learning to speak, and the patient with a moderate phenotype was able to run and ride a bicycle (Hwu *et al*, 2012; Chien *et al*, 2017; Kojima *et al*, 2019).

Efficacy was further supported by positron emission tomography (PET). In all three studies, PET revealed increased putaminal uptake of l‐6‐[^18^F] fluoro‐3, 4‐dihydroxyphenylalanine or 6‐[^18^F]fluoro‐l‐*m*‐tyrosine (FMT) tracers after gene transfer (Hwu *et al*, 2012; Chien *et al*, 2017; Kojima *et al*, 2019). Additionally, CSF analysis showed increased DA and serotonin metabolites (Hwu *et al*, 2012; Chien *et al*, 2017; Kojima *et al*, 2019).

Transient dyskinesia was observed in all patients but resolved within months, and one patient experienced apneic episodes that diminished after ten months (Hwu *et al*, 2012; Chien *et al*, 2017; Kojima *et al*, 2019). Pyrexia was also commonly observed (16%) (Chien *et al*, 2017). One instance of subdural hemorrhage with no clinical symptom was documented (Kojima *et al*, 2019).

These studies together demonstrate that the restoration of DA synthesis in the putamen via gene therapy using low doses of AAV2‐hAADC is well tolerated, leads to sustained improvements in motor and nonmotor symptoms of AADC deficiency, and is overall beneficial for the patients.

## Proposed mechanism of action of intraputaminal AADC gene therapy

Direct transduction of MSNs in the putamen and subsequent production of AADC appears to be the primary mechanism of action (Christine *et al*, 2009). AAV2‐transduced MSNs replace the degenerating nigra afferents in PD or allow the use of endogenous l‐DOPA to synthesize DA in AADC deficiency (Muramatsu *et al*, 2002). DA produced in transduced MSNs could leak into extracellular space and bind DA receptors. Alternatively, DA could activate DA signaling intracellularly. In PD, after systemic levodopa administration, the neutral amino acid transporter is likely responsible for its entry into the MSNs. The mechanism of DA release has not been fully characterized; however, the MSNs of parkinsonian rodents treated with levodopa were shown to release DA in microdialysis experiments (Sánchez‐Pernaute *et al*, 2001).

Intraputaminal administration of viral vectors may also work through other potential mechanisms, either alone or in combination, to increase DA levels in the brain. In addition to MSNs, the striatum contains bienzymatic nondopaminergic neurons that express tyrosine hydroxylase (TH) and AADC and thus can produce DA under normal conditions. The striatum also contains monoenzymatic nondopaminergic neurons that express only TH or AADC. Thus, when a functional AADC‐encoding *DDC* gene is delivered via gene therapy, the TH‐producing monoenzymatic cells could acquire new functionality and begin producing AADC and subsequently DA while the AADC‐producing monoenzymatic cells could become active by gaining a functional *DDC* gene and restore biosynthesis of DA in the CNS (Christine *et al*, 2009; Ugrumov, 2009; Muramatsu *et al*, 2010).

Preclinical studies have indeed demonstrated DA synthesis by striatal monoenzymatic neurons in mice and in a non‐human primate PD model. Additionally, because this class of neurons can release, but not recover DA via reuptake transporters, the extracellular diffusion of excess striatal DA would allow dopaminergic nigrostriatal nerve terminals with uptake transporters to “resupply” synaptic DA and potentially return the circuit to more normal function (Ugrumov, 2009).

In 2021, Onuki *et al* demonstrated that persistent dopaminergic restoration in the prefrontal cortico‐putaminal network is a basis for the therapeutic effects of AADC gene therapy on the motor system (Onuki *et al*, 2021). Neuroimaging data of 8 patients (3 females and 5 males) with AADC deficiency who received gene therapy in an open‐label phase 1/2 study (Kojima *et al*, 2019; Onuki *et al*, 2021) showed increased FMT uptake in the putamen, and motor improvement was associated with dopaminergic restoration of the putaminal area. This clinical evidence suggests that putaminal dopamine promotes the development of the immature motor control system and possibly an internal model for motor control, particularly in the prefrontal cortex, which is primarily affected by AADC deficiency. In contrast, intraputaminal gene therapy in PD causes the functional recovery of a well‐developed motor system (Onuki *et al*, 2021).

## Additional gene therapy targets and potential limitations

Additional sites for AADC gene therapy in PD and AADC deficiency are the dopaminergic areas of the midbrain, the SN, and the ventral tegmental area. As of this writing, no peer‐reviewed literature has been published on the midbrain. However, given that the broad cortico‐putaminal network appears to be involved in motor recovery following intraputaminal infusion of gene therapy, it may not be necessary to target these deeper brain structures, which are smaller than the putamen and more difficult to access surgically (Onuki *et al*, 2021).

To enable a realistic and objective assessment of different therapeutic strategies against AADC deficiency, the clinical course of patients after gene therapy should be documented in a uniform and structured manner in an industry‐independent patient registry. The basis for this could be the registry of the International Working Group on Neurotransmitter Related Disorders (iNTD), which has been collecting data on the natural course, epidemiology, genotype/phenotype correlations, and clinical outcomes of different therapeutic strategies of patients with neurotransmitter disorders from 42 centers in 26 countries since 2013 (Opladen *et al*, 2016).

## Conflict of interest

WLH participated as an advisory board member, received consulting fees, and was a speaker for PTC Therapeutics, BioMarin, and Sanofi; was a grant recipient for PTC Therapeutics and BioMarin; and was research investigator for PTC Therapeutics. SM owns stock in Gene Therapy Research Institution; he participated as an advisor board member, received consulting fees, and was a speaker for PTC Therapeutics. DC is an employee, research investigator, and stock owner for PTC Therapeutics. PP has received consulting fees from PTC Therapeutics and Origin Biotherapeutics. TO was a grant recipient of Dietmar‐Hopp‐Foundation and a speaker for PTC Therapeutics. TR and KK have received consulting fees for PTC Therapeutics. AR has received consulting fees from PTC Therapeutics and GW Pharmaceuticals. IA and TN have no relevant relationships to disclose.
